# The utility of the reperfusion rate of tissue oxygen saturation as a measure of vascular endothelial function in adolescents: reliability, validity and sensitivity

**DOI:** 10.3389/fphys.2023.1163474

**Published:** 2023-09-13

**Authors:** Sascha H. Kranen, Ricardo S. Oliveira, Bert Bond, Craig A. Williams, Alan R. Barker

**Affiliations:** ^1^ Children’s Health and Exercise Research Centre, Public Health and Sports Sciences, University of Exeter Medical School, Faculty of Health and Life Sciences, University of Exeter, Exeter, United Kingdom; ^2^ Department of Physical Education, Federal University of Rio Grande do Norte, Natal, Brazil

**Keywords:** FMD, NIRS, vascular function, repeatability, youth

## Abstract

**Introduction:** The near-infrared spectroscopy (NIRS)-derived reperfusion rate of tissue oxygen saturation (slope 2 StO_2_) may provide a surrogate measure of vascular function, however, this has yet to be examined in a paediatric population. This study investigated in adolescents: 1) the between-day reliability of NIRS-derived measurements; 2) the relationship between slope 2 StO_2_ and macro- (flow-mediated dilation, FMD) and microvascular (peak reactive hyperaemia, PRH) function; and 3) the effect of high-intensity interval exercise (HIIE) on slope 2 StO_2_, FMD, and PRH.

**Methods:** Nineteen boys (13.3 ± 0.5 y) visited the laboratory on two occasions, separated by ∼ 1 week. On visit 1, participants underwent simultaneous assessment of brachial artery FMD and slope 2 StO_2_ and PRH on the internal face of the forearm. On visit 2, participants completed a bout of HIIE with slope 2 StO_2_, FMD and PRH measured pre-, immediately post- and 1.5 h post-exercise.

**Results:** Slope 2 StO_2_ showed no mean bias (*p* = 0.18) and an intraclass correlation coefficient of 0.67 (*p* = 0.003) between visits. No significant correlation between slope 2 StO_2_ and FMD or PRH was observed on visit 1 (*r* = −0.04, *p* = 0.89 and *r* = −0.30, *p* = 0.23, respectively) or visit 2 pre-exercise (*r* = −0.28, *p* = 0.25 and *r* = −0.31, *p* = 0.20, respectively). Compared to pre-exercise, FMD decreased immediately post-exercise (*p* < 0.001) and then increased 1.5 h post-exercise (*p* < 0.001). No significant change was detected for slope 2 StO_2_ (*p* = 0.30) or PRH (*p* = 0.55) following HIIE.

**Conclusion:** In adolescents, slope 2 StO_2_ can be measured reliably, however, it is not correlated with FMD or PRH and does not follow the acute time course of changes in FMD post-exercise. Hence, the use of slope 2 StO_2_ as a surrogate measure of vascular function in youth must be refuted.

## Introduction

Endothelial dysfunction is the first step in the development of atherosclerosis, a precursor to overt cardiovascular disease (CVD), and has been observed in adolescents with CVD risk factors (Celermajer et al., 1992). The assessment of vascular function using flow-mediated dilation (FMD) is popular due to its non-invasive nature (Thijssen et al., 2019). However, the FMD procedure is technically challenging and the analysis of FMD data is time-consuming. Therefore, it is of great interest to establish further non-invasive approaches to assess vascular endothelial function in youth.

In adults, some studies have employed near-infrared spectroscopy (NIRS)-derived measurements of tissue oxygen saturation, especially the reperfusion rate of tissue oxygen saturation (slope 2 StO_2_), after a period of blood flow occlusion, to assess microvascular function (Gerovasili et al., 2010; Willingham et al., 2016). Several studies have shown that the measurement of slope 2 StO_2_ is reliable in adults. For instance, McLay et al. (2016b) have demonstrated good within-day and between-day repeatability of slope 2 StO_2_ in the leg. Iannetta et al. (2019) tested the reliability of between-day assessments of slope 2 StO_2_ across different occlusion periods. The authors reported good repeatability with most reliable measurements observed following 5 min of occlusion (ICC of 0.88), which is consistent with the commonly used period of ischaemia for the FMD assessment (Thijssen et al., 2019), demonstrating the measurements can be undertaken simultaneously. Previous research has shown that the slope 2 StO_2_ is significantly correlated (*r* = 0.63, *p* = 0.003) with FMD in the leg in healthy young adults (McLay et al., 2016a). More recently, Soares et al. (2019b) measured brachial artery FMD and slope 2 StO_2_ in the forearm in healthy adults and reported a significant correlation between these parameters (*r* = 0.66; *p* = 0.001).

Given the above, the NIRS-derived slope 2 StO_2_ may be used as a cheaper, operator-independent, and less labour-intensive surrogate of vascular endothelial function. However, it has been proposed that the observed correlation between slope 2 StO_2_ and FMD is driven by the relationship between slope 2 StO_2_ and shear rate in the conduit artery (Tremblay and King, 2016). While shear rate and FMD are related in adults (Thijssen et al., 2009; Thijssen et al., 2019), this relationship is not present in youth (Thijssen et al., 2009; Bond et al., 2015a; Bond et al., 2015b; Kranen et al., 2019; Kranen et al., 2021), suggesting that slope 2 StO_2_ and FMD may not be significantly correlated in this population. Presently, no study has examined whether the reliability and validity of NIRS-derived slope 2 StO_2_ hold true for a paediatric population. Confirmation of the previous reports regarding slope 2 StO_2_ and its correlation with FMD would enable the execution of large cohort studies of vascular function in a youth population.

Studies have shown that slope 2 StO_2_ can differentiate between young and elderly adults (Rosenberry et al., 2018) and trained and untrained individuals (Soares et al., 2018a) and may therefore also be sensitive to an acute exercise stimulus. During exercise, Dawson et al. (2013) proposed the existence of a biphasic FMD pattern in response to an acute bout of high-intensity interval exercise, whereby a significant reduction in FMD immediately upon termination of the exercise is followed by an improvement in FMD beyond baseline values (“supranormal”) 1 h or longer after the exercise bout. This phenomenon has been shown in adults (Birk et al., 2013) and adolescents (Bond et al., 2015b). In contrast, Bond et al. (2015b) reported that microvascular function in the form of PRH improved immediately upon exercise cessation and remained elevated for 2 h, suggesting a different time course of response. To our knowledge, no study has examined the effect of exercise on NIRS-derived slope 2 StO_2_ in youth and how this compares to FMD and PRH.

The aims of this study were to address the following in a paediatric population: 1) the between-day reliability of NIRS-derived measurements; 2) examine the relationship between NIRS-derived slope 2 StO_2_ and FMD and PRH, respectively; and 3) to investigate the effect of an acute bout of high-intensity interval running on NIRS-derived slope 2 StO_2_ and compare it to the FMD and PRH response. It was hypothesized that the NIRS-derived measurements would be reliable between days but that there would be no significant association between slope 2 StO_2_ and FMD. In addition, it was hypothesized that FMD followed a biphasic response post-exercise whereas the NIRS-derived slope 2 StO_2_ exhibited an immediate increase which remained up to 90 min post-exercise in accordance with PRH.

## Methods

### Ethics approval

The study conformed to the standards established by the Declaration of Helsinki and was approved by the Sport and Health Sciences Ethics Committee, University of Exeter (161207/B/02). The study was not registered in a database. Before commencement of the project, details of the study and associated risks and benefits were explained, and written participant assent and parental consent were obtained.

### Experimental design

A convenience sample of nineteen 12- to 14-year-old male adolescents from a local secondary school volunteered to participate in research investigating the effect of 4 weeks of high-intensity interval training on cardiovascular health (Kranen et al., 2023). Data for the current investigation was collected as baseline data of this training study. This consisted of two visits in the laboratories at the University of Exeter, separated by ∼ 1 week. With parental supervision, participants were asked to replicate their evening meal prior to each laboratory visit and were instructed to avoid strenuous exercise during the 48 h before each visit. On visit 1, anthropometric and baseline measurements of macro- (FMD) and microvascular function (PRH) and tissue oxygen saturation using NIRS were taken, followed by a fitness assessment. Visit 2 consisted of a replication of the baseline measurements of vascular function and tissue oxygen saturation, a bout of high-intensity interval running followed by further assessments of macro- and microvascular function and tissue oxygen saturation immediately and 1.5 h post-exercise.

#### Visit 1: anthropometric and fitness assessment

Participants were transported by car to the laboratory at 08:00 after an approximately 12 h overnight fast. Upon arrival, anthropometric measurements were taken using standard procedures. Stature and body mass were measured to the nearest 0.1 cm and 0.1 kg, respectively, and body volume was measured using the BodPod (Body Composition System, Life Measurement Instruments, Concord, CA, United States) for the estimation of fat mass and fat free mass which has been validated in this population (Ferri-Morales et al., 2018). Age and sex specific body mass index (BMI) cut-points were consulted to classify participants as overweight or obese (Cole et al., 2000). Pubertal status was estimated through self-assessment of secondary sexual characteristics using adapted drawings of the five stages of pubic hair development (Morris and Udry, 1980). Fingertip capillary blood samples were then taken for the analysis of glucose, insulin, cholesterol and blood lipids.

**FIGURE 1:**
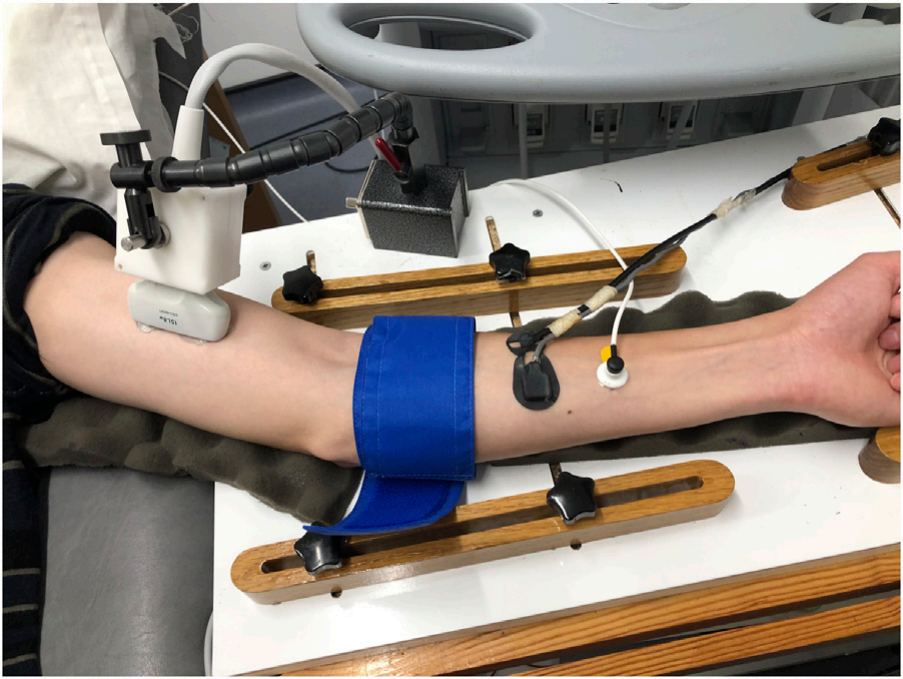
Representative image of placement of different probes and pneumatic cuff on the left arm. From left to right: ultrasound probe, pneumatic cuff, NIRS probe, laser Doppler probe.

At ∼ 08:30, participants rested in a supine position in a darkened, temperature-controlled room (24°C) for ∼10 min before the simultaneous assessment of macrovascular (FMD), microvascular (laser Doppler Perfusion monitoring) functions (Bond et al., 2017) and tissue oxygen saturation using NIRS (see Figure 1). After these measurements, participants walked from the laboratory (∼1 min) to the adjacent sports hall and performed a multistage 20 m shuttle run test (Leger et al., 1988) to determine their maximal aerobic speed. This test is both a reliable and valid measure of maximal aerobic speed (MAS) in adolescents (Liu et al., 1992). Participants were asked to run between two lines set 20 m apart by following the pace of an audio signal produced from a CD player. The test began at a speed of 8.5 km∙h^−1^ and increased by 0.5 km∙h^−1^ each minute until volitional exhaustion which was assumed when participants were not able to reach the line in the required time frame on two occasions. The speed of the last complete shuttle run was taken as MAS. Cardiorespiratory fitness was then classified according to age- and sex-specific normative values using the speed at the last complete stage (Tomkinson et al., 2017).

#### Visit 2: high-intensity interval exercise bout

In accordance with visit 1, participants were transported to the laboratory at 08:00 by car after an overnight fast of at least 12 h. Following arrival, vascular function and tissue oxygen saturation were assessed simultaneously. The locations of the different probes during the first scan (pre-exercise) were marked on the arm with a pen for replication in later measurements. Subsequently, participants walked to the same adjacent sports hall and performed a bout of high-intensity interval running. Following a 1 min warm-up of jogging at 6 km·h^−1^, participants performed 8 × 1 min running intervals at 90% of MAS, interspersed with 75 s of passive rest. Participants completed the intervals by running back and forth between two cones following an audio signal. A cone was placed at either side of the sports hall and the distance between them was determined individually for each participant so that the speed required to cover the distance within the time frame equated to 90% MAS. Heart rate was measured throughout the exercise bout using short-range telemetry (Polar Team2, Polar Electro, Kempele, Finland). During the recovery period participants rested upright near the starting cone.

Immediately upon completion of the exercise bout participants walked back to the laboratory (∼1 min) and underwent another simultaneous assessment of vascular function and tissue oxygen saturation. Subsequently, participants pursued sedentary activities (e.g., playing board games, watching films, etc.) for ∼1 h before a final measurement of vascular function and tissue oxygen saturation took place 90 min after the exercise bout.

### Macrovascular function

Macrovascular function was assessed in the brachial artery of the left arm. High resolution Doppler and B-mode images of the brachial artery were simultaneously assessed (Sequoia 512, Acuson, Siemens Corp, Aspen, CO, United States) with a 13 MHz linear array transducer, in accordance with current guidelines (Thijssen et al., 2019) and our earlier work (Bond et al., 2017). Following a ∼10 min acclimatization period to the temperature-controlled room (24°C) in the supine position, baseline arterial diameter was measured for 1 min. Endothelium-dependent vasodilation of the brachial artery was measured for 3 min after a 5 min ischaemic stimulus induced by rapid forearm pneumatic cuff inflation (moorVMS-PRES, Moor Instruments Ltd., Axminster, United Kingdom) to 200 mmHg. Baseline arterial diameter and post-occlusion brachial artery diameter were assessed during end diastole using validated ECG-gating software (Medical Imaging Applications LLC, Coralville, IA, United States) (Thijssen et al., 2019). FMD was calculated using the following equation:

$$\text{FMD }\left(\%\right)=\frac{Peak\;post-occlusion\;diameter-Mean\;baseline\;diameter}{Mean\;baseline\;diameter}\;x\;100\%$$

All analyses were performed by the same investigator. The area under the curve for estimated shear rate (SR_AUC_) was calculated from the time of cuff deflation until peak dilation (Pyke and Tschakovsky, 2005). In accordance with other paediatric data reported by our laboratory (Bond et al., 2015a; Bond et al., 2015b; Kranen et al., 2019; Kranen et al., 2021) and others (Thijssen et al., 2009), preliminary analyses using Pearson’s correlation coefficient (*r*) revealed the absence of significant correlations between SR_AUC_ and FMD. Consequently, FMD was not normalised for shear, however, shear data are presented separately in compliance with the current reporting guidelines (Thijssen et al., 2019). Due to the suggestion by Atkinson and Batterham (2013) to adjust FMD allometrically for baseline diameter, Pearson’s correlation coefficient (*r*) was used to examine the relationship between FMD and baseline diameter. However, as no consistently significant correlations between FMD and baseline diameter were detected, allometric scaling was not performed.

### Microvascular function

Microvascular function was assessed simultaneously to the FMD protocol using a laser Doppler perfusion monitor (moorVMS-LDF, Moor Instruments Ltd., Axminster, United Kingdom), which was calibrated according to the manufacturer’s guidelines. An optic probe with 8 collecting fibres in a 2 mm ring with a central delivery fibre was attached using adhesive stickers to a reproducible point on the distal third of the forearm (Cracowski et al., 2006). High resolution data were collected at 40 Hz. Outcome measure were peak reactive hyperaemia (PRH), which was defined as the highest point after cuff deflation in relation to the baseline average and expressed as the difference between the two measures, and time to PRH (PRH_t_).

### Near-infrared spectroscopy

Tissue oxygen saturation was measured using near-infrared spectroscopy (NIRO−200, Hamamatsu Photonics Deutschland GmbH, Herrsching am Ammersee, Germany) with four laser diodes which emit light at different wavelengths (776, 826, 845, and 905 nm). Both the emitter and detector were sheathed in a rubber holder with 4 cm spacing and attached to the distal third of the forearm at a reproducible point ∼5 cm proximal to the laser Doppler probe using a double-sided adhesive sticker. Oxygenated (HbO_2_) and deoxygenated (HHb) haemoglobin were continuously determined during the FMD protocol at a frequency of 1 Hz. Tissue oxygen saturation (StO_2_) was estimated as (HbO_2_)/[(HbO_2_ + HHb)] and expressed as a percentage. In line with McLay et al. (2016a), the following parameters were derived from the NIRS measurement: StO_2_ baseline (%) was calculated as the average StO_2_ during the 1 min period before cuff occlusion. The nadir reached during the last 30 s of ischaemia was defined as StO_2_ minimum (%). The desaturation rate of tissue oxygen saturation was quantified as the downslope of StO_2_ during ischaemia (StO_2_ slope 1, %·s^−1^). The StO_2_ reperfusion rate was calculated using the upslope of the StO_2_ signal in the first 10 s following cuff release (StO_2_ slope 2, %·s^−1^). StO_2_ peak (%) was defined as the highest StO_2_ value achieved after cuff release.

### Blood analyses

All blood samples were analysed in duplicate and the mean was used for subsequent analyses. One blood sample was analysed for total cholesterol, high-density lipoprotein (HDL), low-density lipoprotein (LDL) and triacylglycerol (TAG) (CardioChek PA, BHR Pharmaceuticals Ltd., Nuneaton, United Kingdom). Two further fingertip capillary blood samples (∼200 µL each) were taken into heparin/fluoride coated microvettes (CB 300 FH tubes, Sarstedt AG and Co., Nümbrecht, Germany). One sample was analysed immediately for blood (glucose) (YSI 2900D Biochemistry Analyzer, YSI Inc., Yellow Springs, OH, United States) while the other was centrifuged at 4,000 revolutions per minute for 8 min. Then plasma was separated from the centrifuged sample and stored at −80°C for later analysis of plasma [insulin]. Plasma [insulin] was measured in duplicate by enzyme immunoassay (DRG Instruments GmbH, Marburg, Germany). Absorbance at 450 nm, reference 620 nm, was recorded using an EnSpire 2,300 plate reader (Perkin Elmer Inc., Waltham, MA, United States). Values were quantified against a 5−parameter standard curve (0, 6.25, 12.5, 25, 50, and 100 µLU/mL). The within-batch coefficient of variation for plasma [insulin] analysis was 5.6%.

### Statistical analyses

All data are presented as mean and standard deviation (SD) unless otherwise stated. Descriptive statistics were used to analyse participant characteristics and details from both fitness assessment and high-intensity exercise bout. The reliability of the vascular and NIRS-derived measurements was examined using the typical error (TE), the TE expressed as a coefficient of variation (CV) and the intraclass correlation coefficient (ICC) (Hopkins, 2000). The level of reliability was classified according to Portney and Watkins (2009) with ICC values >0.90 as excellent, values between 0.75 and 0.90 as good, values between 0.5 and 0.75 as moderate and values <0.5 as poor reliability. Pearson’s correlation coefficient (*r*) was employed for the analysis of the relationship between slope 2 StO_2_ and FMD and PRH, respectively, and the correlation between shear rate and slope 2 StO_2_. A repeated measures ANOVA with time as independent variable (Pre, Post, 1.5 h Post) was used to examine the vascular and NIRS-derived responses following the exercise bout. Significant main effects were further analysed using paired samples t-tests and interpreted using the *p*-value and standard effect sizes (ES), with the latter used to determine the magnitude of the observed effect according to the following grading: small (0.2), moderate (0.5), and large (0.8) (Cohen, 1988). Statistical significance was accepted when *p* < 0.05. Sample size was calculated *a priori* using G*Power aiming to detect a correlation coefficient of *r* = 0.63 observed by McLay et al. (2016a). With the inclusion of statistical power of 80% and an alpha level of 0.05, a total sample size of 17 participants was required.

IBM SPSS Statistics software (Version 25; IBM Corporation, Armonk, NY, United States) was used for all statistical analyses.

## Results

Characteristics for the nineteen participants are presented in Table 1. One participant was categorized as overweight. Maturity status for the adolescent boys was as follows: stage 1, *n* = 1, stage 2, *n* = 5, stage 3, *n* = 5, stage 4, *n* = 7, respectively. One participant declined the self-assessment of maturity status. Participants were ranked in the following percentiles for their cardiorespiratory fitness: 20th, *n* = 1, 60th, *n* = 3, 70th, *n* = 6, 80th, *n* = 4 and 90th, *n* = 5.

**TABLE 1:** Participant descriptive characteristics.

	Mean ± SD	Minimum	Maximum
Age (y)	13.3 ± 0.5	12.7	14.6
Stature (m)	1.62 ± 0.09	1.48	1.77
Body mass (kg)	47.1 ± 7.9	36.6	68.1
BMI (kg∙m^−2^)	18.0 ± 1.9	15.1	22.5
Fat mass (kg)	9.3 ± 4.2	4.7	19.5
Fat free mass (kg)	37.7 ± 6.9	27.8	50.7
Body fat (%)	19.7 ± 7.4	8.4	35.9
Blood glucose (mmol∙L^−1^)	5.63 ± 0.40	4.61	6.01
Total cholesterol (mmol∙L^−1^)	3.42 ± 0.45	2.68	4.36
HDL (mmol∙L^−1^)	1.36 ± 0.25	1.02	1.97
LDL (mmol∙L^−1^)	1.92 ± 0.51	1.11	2.84
TAG (mmol∙L^−1^)	0.71 ± 0.22	0.57	1.49
Insulin (µLU·mL^−1^)	10.42 ± 3.52	4.78	18.52

BMI, body mass index; HDL, high-density lipoprotein; LDL, low-density lipoprotein; TAG, triacylglycerol.

Due to a technical failure of the pneumatic cuff on visit 1, one participant had to be excluded from both the reliability and the correlation analysis between slope 2 StO_2_ and FMD and PRH.

**FIGURE 2:**
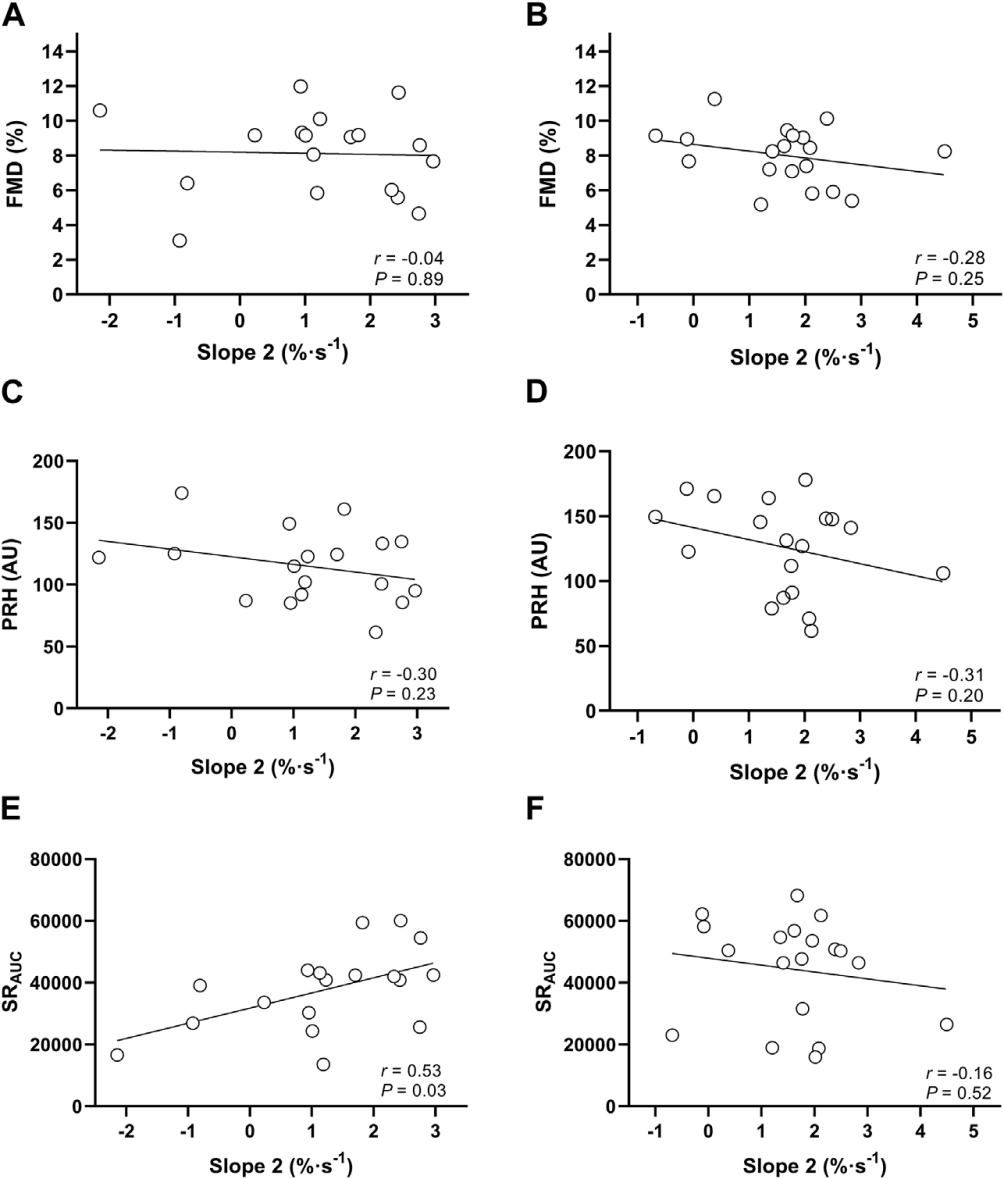
Relationship between flow-mediated dilation (FMD) and the reperfusion rate of tissue oxygen saturation (Slope 2) on visit 1 **(A)** and visit 2 **(B)**; relationship between peak reactive hyperaemia (PRH) and Slope 2 on visit 1 **(C)** and visit 2 **(D)**; and relationship between shear rate and slope 2 StO_2_ on visit 1 **(E)** and visit 2 **(F)**.

An overview of all vascular and NIRS-derived measurements is presented in Table 2. Analyses revealed ICCs of 0.67 for slope 2 StO_2_, 0.68 for FMD and 0.23 for PRH between visits. The reproducibility of all measurements is illustrated in Table 3. There was no significant correlation between StO_2_ slope 2 and FMD or between StO_2_ slope 2 and PRH on visit 1 or visit 2 (all *p* > 0.19). However, shear rate and slope 2 StO_2_ were significantly correlated on visit 1 (*r* = 0.53, *p* = 0.025) but not on visit 2 (*r* = −0.16, *p* = 0.52). See Figure 2 for details.

**TABLE 2:** Macro- and microvascular and NIRS measurements.

	Visit 1	Visit 2	Visit 2	Visit 2
	Baseline	Baseline	Post-exercise	1.5 h post-exercise
FMD				
Baseline diameter (mm)	2.90 ± 0.40	2.94 ± 0.38	3.04 ± 0.29	2.93 ± 0.37^#^
Peak diameter (mm)	3.13 ± 0.39	3.18 ± 0.40	3.21 ± 0.31	3.23 ± 0.40
FMD (%)	8.12 ± 2.42	8.01 ± 1.64	5.47 ± 1.89^*^	10.48 ± 2.37^*#^
Shear AUC (AU)	37,776 ± 13,175	44,333 ± 16,515	73,508 ± 30,442^*^	56,536 ± 16,370^*#^
Microvascular				
PRH (AU)	115.0 ± 29.2	126.3 ± 35.5	124.9 ± 58.7	135.3 ± 44.5
PRH_t_ (s)	13.7 ± 7.6	18.9 ± 13.3	16.6 ± 13.2	21.6 ± 12.2
NIRS				
StO_2_ baseline (%)	62.8 ± 6.3	62.7 ± 5.5	58.6 ± 6.1^*^	61.8 ± 5.7^#^
StO_2_ minimum (%)	45.3 ± 8.8	41.2 ± 8.3	37.8 ± 7.7^*^	41.2 ± 7.8^#^
StO_2_ slope 1 (%·s^−1^)	−0.05 ± 0.04	−0.07 ± 0.04	−0.06 ± 0.03	−0.06 ± 0.04
StO_2_ peak (%)	69.1 ± 6.7	69.7 ± 6.5	68.3 ± 7.6^*^	69.6 ± 7.4
StO_2_ slope 2 (%·s^−1^)	1.22 ± 1.41	1.61 ± 1.18	1.73 ± 1.16	1.53 ± 1.09

Values are means ± SD. FMD, flow-mediated dilation; SR_AUC_, shear rate area under the curve; StO_2_, tissue oxygen saturation; Slope 1 StO_2_, desaturation rate of tissue oxygen saturation; Slope 2 StO_2_, reperfusion rate of tissue oxygen saturation; PRH, peak reactive hyperaemia; PRH_t_, time to peak reactive hyperaemia. * sign. difference (*p* < 0.05) from pre-exercise measurement. # sign. difference (*p* < 0.05) from post-exercise measurement.

**TABLE 3:** Between-day reproducibility of macro- and microvascular and NIRS measurements.

	Change in mean between visits	*p*-Value	Typical error	Typical error as CV (%)	ICC
FMD					
Baseline diameter (mm)	0.05	0.17	0.10	3.5	0.94
Peak diameter (mm)	0.05	0.11	0.10	3.0	0.95
FMD (%)	−0.13	0.75	1.22	20.3	0.68
SR_AUC_ for FMD (AU)	7,977	0.11	14,312	45.7	0.03
Microvascular					
PRH (AU)	14.4	0.14	27.9	29.6	0.23
NIRS					
Baseline StO_2_ (%)	−0.05	0.97	3.89	6.7	0.60
Minimum StO_2_ (%)	−3.74	0.10	6.53	17.8	0.45
Slope 1 StO_2_ (%·s^−1^)	−0.02	0.22	1.15	#	0.45
Peak StO_2_ (%)	0.72	0.63	0.86	7.0	0.60
Slope 2 StO_2_ (%·s^−1^)	0.37	0.18	0.78	#	0.67

CV, coefficient of variation; ICC, intraclass correlation coefficient; FMD, flow-mediated dilation; SR_AUC_, shear rate area under the curve; StO_2_, tissue oxygen saturation; Slope 1 StO_2_, desaturation rate of tissue oxygen saturation; Slope 2 StO_2_, reperfusion rate of tissue oxygen saturation; PRH, peak reactive hyperaemia. # Negative values did not allow a loglinear transformation for the calculation of the typical error as CV (%).

**FIGURE 3:**
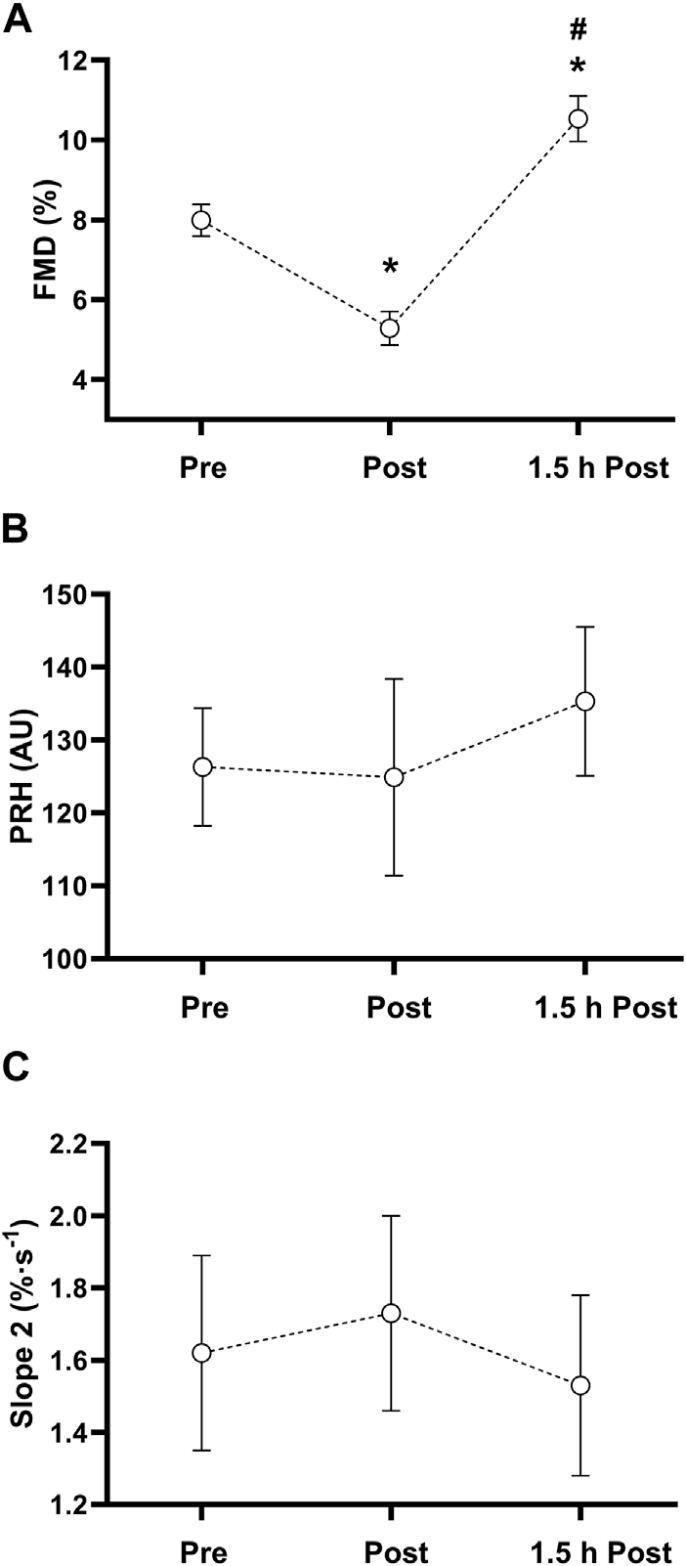
Macro- and microvascular and tissue oxygen saturation responses before (Pre), immediately after (Post) and 1.5 h after (1.5 h Post) a bout of high-intensity interval exercise. **(A)** Flow-mediated dilation (FMD) **(B)** Peak reactive hyperaemia (PRH) **(C)** Reperfusion rate of tissue oxygen saturation (Slope 2). * sign. difference (*p* < 0.001) from pre-exercise measurement. # sign. difference (*p* < 0.001) from post-exercise measurement.

Participants achieved a MAS of 12.0 ± 0.7 km·h^−1^ with a maximum heart rate of 202 ± 7 b·min^−1^ in the shuttle run test. A summary of the results of the fitness assessment and the high-intensity interval exercise bout is provided in Table 4. Vascular and NIRS-derived responses to the exercise bout are depicted in Figure 3. The ANOVA revealed a significant main effect of time for FMD (*p* < 0.001). FMD was significantly reduced (*p* < 0.001, ES = 1.44) immediately after the exercise bout. Subsequently, FMD increased significantly 1.5 h after cessation of the acute exercise (*p* < 0.001, ES = 2.34). FMD was also significantly greater 1.5 h post-exercise compared to pre-exercise (*p* < 0.001, ES = 1.21). No significant main effect of time was detected for PRH (*p* = 0.55) or slope 2 StO_2_ (*p* = 0.30) in response to the bout of high-intensity interval exercise.

**TABLE 4:** Details of fitness assessment and high-intensity exercise bout.

	Mean ± SD	Minimum	Maximum
Shuttle run test			
MAS (km·h^−1^)	12.0 ± 0.7	10.0	13.0
HR_max_ (b·min^−1^)	202 ± 7	190	216
High-intensity bout			
Interval speed (km·h^−1^)	10.8 ± 0.6	9.0	11.7
Average HR (b·min^−1^)	160 ± 12	135	181
Average HR (%HR_max_)	79 ± 5	70	88
Peak HR (b·min^−1^)	200 ± 8	181	208
Peak HR (%HR_max_)	98 ± 3	94	105

MAS, maximal aerobic speed; HR, heart rate; HR_max_, maximum heart rate.

## Discussion

This is the first study to comprehensively assess the between-day repeatability of NIRS-derived measurements, examine the relationship between NIRS-derived slope 2 StO_2_ and FMD and PRH, respectively, and investigate the effect of an acute exercise bout on slope 2 StO_2_ in a paediatric group. The main finding of the current study was that slope 2 StO_2_ can be measured reliably between days in this population and its repeatability is comparable to that of FMD but superior to PRH. A further novel finding was that, in adolescents, slope 2 StO_2_ is not significantly correlated with FMD and PRH. Finally, slope 2 StO_2_ and PRH did not track the classic biphasic time course for FMD (Dawson et al., 2013) in response to an acute bout of high-intensity interval exercise. Consequently, the use of slope 2 StO_2_ as a surrogate for FMD in adolescents must be refuted.

### Between day reliability of slope 2 StO_2_

In the current study the between day reliability of brachial artery FMD and slope 2 StO_2_ assessed on the forearm were strikingly similar (ICC of 0.68 and 0.67, respectively). Previously, McLay et al. (2016b) reported weaker reliability for FMD than slope 2 StO_2_ (ICC of 0.25 and 0.94, respectively). However, their measurement of FMD did not conform to the guidelines (Thijssen et al., 2019) which may have contributed to the poor reliability. Although artery diameter was captured continuously after cuff deflation, the diameter was only analysed every 15 s. It is highly likely that the true peak diameter may have been missed and that this procedure has confounded the FMD reliability due to the variation in the time to peak (Black et al., 2008). Furthermore, McLay et al. (2016b) assessed vascular function in the popliteal artery whereas FMD is commonly measured in the brachial artery (Raitakari and Celermajer, 2000; Thijssen et al., 2019). Others have shown that the assessment of FMD is highly reproducible in multicentre studies in healthy adult participants with a coefficient of variation (CV) of 11.6%–16.1% (Ghiadoni et al., 2012) and in paediatric populations as shown here (CV of 20.3%) and in previous studies (Bond et al., 2017; Kranen et al., 2019) when adhering to the guidelines (Thijssen et al., 2019). Iannetta et al. (2019) examined the reliability of slope 2 StO_2_ on the tibialis anterior muscle in younger and older adults and presented a higher ICC of 0.88 with no differences between age groups. Apart from the difference in the sample population, the aforementioned study measured slope 2 StO_2_ in the leg microvasculature.

Unlike FMD and slope 2 StO_2_, PRH had an ICC of only 0.23 in the current study, which is rated as “poor.” In comparison, Bond et al. (2017) reported a superior ICC of 0.54 for PRH in adolescents. The difference in reproducibility of PRH between studies may be explained by the use of different equipment. Additionally, despite best efforts to attach the probe to the same location on the arm, it cannot be ruled out that slight differences in position may have negatively impacted the reproducibility of PRH. Akin to the current observation, Roustit et al. (2010) reported poor reproducibility of PORH (ICC of 0.29) on the forearm and suggested spatial variability as a reason.

### Criterion validity of slope 2 StO_2_

In the present study we observed no significant correlation between slope 2 StO_2_ and FMD on either visit. This finding contrasts with the original findings of McLay et al. (2016a) who reported that slope 2 StO_2_ and FMD are significantly correlated in the leg (*r* = 0.63, *p* = 0.003). Soares et al. (2019b) later assessed brachial artery FMD and the NIRS-derived slope 2 StO_2_ on the forearm in healthy young men. The authors reported a significant correlation between these two measures (*r* = 0.66; *p* = 0.001), which contrasts with our non-significant findings. Collectively, these studies show good agreement between slope 2 StO_2_ and FMD across limbs in adults. However, Tremblay and King (2016) argued against the use of slope 2 StO_2_ as a measure of macrovascular function because it is not a direct reflection of FMD but of its stimulus, i.e., the shear rate in the artery. Slope 2 StO_2_ is reliant on microvascular reactivity that in turn is responsible for downstream resistance for flow and shear rate (Tremblay and King, 2016). Therefore, the authors hypothesized that slope 2 StO_2_ would be better associated with shear rate than FMD. As it has previously been shown that shear rate and FMD are significantly correlated in adults (Thijssen et al., 2009), it is coherent that FMD and NIRS-derived slope 2 StO_2_ are then also correlated. Soares et al. (2019b) were unable to present shear rate data because their ultrasound system precluded the simultaneous assessment of artery diameter and blood velocity. Similarly, McLay et al. (2016a) failed to measure shear rate, so it can only be speculated that shear rate was correlated with FMD, which then drove the relation between FMD and slope 2 StO_2_ in these studies. In the present study, shear rate and FMD were not correlated which is in agreement with other data in paediatric groups (Thijssen et al., 2009; Bond et al., 2015a; Bond et al., 2015b; Kranen et al., 2019; Kranen et al., 2021) and may explain the lack of a significant correlation between slope 2 StO_2_ and FMD in this investigation. Furthermore, the significant correlation between shear rate and slope 2 StO_2_ on visit 1 supports the theory by Tremblay and King (2016) that slope 2 StO_2_ reflects the shear rate in the artery rather than the FMD response. This correlation was absent on the second visit. However, shear rate data are very variable as evidenced by a CV of 45.7% in the present study and further research is required to elucidate whether slope 2 StO_2_ reflects the shear rate in the artery post-ischaemia.

The absence of a significant correlation between FMD and slope 2 StO_2_ in the current study can also be explained from a mechanistic point of view. Previously, it was demonstrated that there are no strong correlations between macro- and microvascular measurements (Dhindsa et al., 2008) and it is generally acknowledged that the FMD response is NO-mediated (Green et al., 2014). Conversely, the NIRS-derived slope 2 StO_2_ was initially used as a measure of microvascular function (Gerovasili et al., 2010; Willingham et al., 2016; Rosenberry et al., 2018). Wong et al. (2003) showed that the infusion of N^G^-nitro-L-arginine methyl ester to inhibit NO synthase did not change PRH and concluded that NO is not mediating microvascular reactivity. Instead, the microvascular hyperaemic response is partly mediated by prostanoids (Binggeli et al., 2003). Furthermore, FMD and PRH are not strongly correlated (Dhindsa et al., 2008). However, as slope 2 StO_2_ was not significantly correlated with the microvascular outcome of PRH either in the current study, it is proposed that slope 2 StO_2_ and PRH provide different information regarding microvascular function.

### Effect of exercise on slope 2 StO_2_

Following the acute bout of exercise, FMD was altered immediately and 1.5 h post-exercise compared to pre-exercise values. In contrast, slope 2 StO_2_ and PRH remained unchanged post-exercise. These findings reinforce the notion that slope 2 StO_2_ cannot be used as a surrogate measure for FMD.

To our knowledge, this is the first study to show the biphasic response for FMD (Dawson et al., 2013) following a bout of high-intensity interval running in adolescents. Previously, Bond et al. (2015b) showed the existence of a biphasic FMD response after high-intensity exercise in adolescents, however, the exercise modality in their study was cycling. The significant changes immediate and 1.5 h post-exercise confirm the sensitivity of the brachial artery FMD assessment in response to a running intervention. In comparison, we did not observe any changes in slope 2 StO_2_ post-exercise, denying this measurement its sensitivity. Previously, it was shown that the NIRS-derived slope 2 StO2 can detect differences in lower limb vascular responsiveness in obese compared to lean young adults (Soares and Murias, 2018) and age-related impairments in microvascular function when not controlling for tissue ischaemia (Rosenberry et al., 2018).

With regards to exercise training, Soares et al. (2018a) demonstrated that the slope 2 StO_2_ is steeper in the leg of trained than in untrained participants. The same research team also reported increases in slope 2 StO_2_ following the ingestion of 75 g glucose in healthy and obese adults (Soares et al., 2017; Soares et al., 2018b). Taken together, the aforementioned studies indicate that the measurement of slope 2 StO_2_ is sensitive to detect differences between training status and the effect of an acute glucose load in adults. However, this sensitivity was absent in the current study which may be explained by differences between the studies. First of all, the other studies were exclusively concerned with adult populations in contrast to the ostensibly healthy adolescent boys in our investigation. Secondly, to our knowledge, this is the first study that utilised an acute exercise intervention to assess the sensitivity of the NIRS-derived measurement whereas previous studies used slope 2 StO_2_ to describe various conditions in populations (e.g., lean vs. obese, young vs. old, trained vs. untrained) or the effect of an acute glucose load. Furthermore, the site of measurement might be important in such a way that slope 2 StO_2_ is less responsive in the arm. This assumption is supported by the findings of Soares et al. (2018b) who observed a significant difference in slope 2 StO_2_ when measured in the leg versus the arm of trained and untrained individuals. Further endorsement is given by West et al. (2019) who reported, similar to our observation, no changes in slope 2 StO_2_ assessed on the forearm in the 2 hours following the ingestion of either 50 g sucrose with or without 160 mg vitamin C in a sample of healthy adults which would reasonably be expected to alter FMD (Loader et al., 2017). Another potential confounder for the deviating results for FMD and slope 2 StO_2_ are the underlying mechanisms detailed above (NO-mediated versus not NO-mediated).

Similar to slope 2 StO_2_, no differences were observed in PRH following exercise which is in contrast to previous investigations reporting an increase after a bout of high-intensity interval exercise (Bond et al., 2015a; Bond et al., 2015b; Kranen et al., 2021). However, this disparity may be attributed to a weaker reliability of the measurement. The other studies reported between-day coefficients of variation of 16.2% (Bond et al., 2015a), 13.3% (Bond et al., 2015b) and 19.4% (Kranen et al., 2021). In comparison, the between-day coefficient of variation in the present investigation was 29.6%, which impedes the finding of changes.

### Considerations and limitations

This is the first study to examine the utility of the slope 2 StO_2_ as a measure of vascular endothelial function in healthy youth. There are several strengths to this study, including the simultaneous assessment of the NIRS-derived oxygen saturation parameters and both macro- and microvascular function in line with recent research and guidelines (Cracowski et al., 2006; McLay et al., 2016a; Thijssen et al., 2019), the individualised high-intensity exercise bout and the multiple assessments post-exercise. However, this study also has limitations. One obvious limitation is the lack of female participants in the study. However, it was previously reported that there were no significant differences in vascular function between sexes at rest or following an acute bout of exercise (Bond et al., 2015b). Furthermore, the NIRS-derived StO_2_ parameters were determined on the forearm whereas in the original paper, which prompted this study, StO_2_ was assessed in the tibialis anterior muscle (McLay et al., 2016a). This may be an important difference because the hyperaemic response is significantly greater following forearm occlusion compared to an occlusion of the lower limb (Soares et al., 2019a). However, Soares et al. (2019b) published data on brachial FMD and slope 2 StO_2_ assessed on the forearm that supported the previous findings by McLay et al. (2016a), potentially rendering the aforementioned difference irrelevant.

## Conclusion

Both FMD and slope 2 StO_2_ can be measured reliably in the brachial artery and the internal face of the forearm, respectively, in adolescent boys. Although the reliability of FMD and slope 2 StO_2_ are comparable, the use of slope 2 StO_2_ as a surrogate for FMD cannot be advocated due to a lack of significant correlation between FMD and slope 2 StO_2_ and importantly the different responses to an acute bout of exercise. Nonetheless, the additional assessment of slope 2 StO_2_ may complement the information gained from the FMD assessment, however, further mechanistic studies are required to elucidate this. This notion is supported by the finding that despite the supranormal FMD response 1.5 h post-exercise, no changes were observed in slope 2 StO_2_ after a bout of high-intensity interval exercise.

## Data Availability

The raw data supporting the conclusion of this article will be made available by the authors, without undue reservation.
